# Factors influencing pocket closure in surgically-treated intraosseous defects. A retrospective analysis

**DOI:** 10.1007/s00784-025-06396-0

**Published:** 2025-05-28

**Authors:** Anna Simonelli, Roberto Farina, Luigi Minenna, Chiara Scapoli, Leonardo Trombelli

**Affiliations:** 1 Research Centre for the Study of Periodontal and Peri-Implant Diseases, University of Ferrara, Corso Giovecca 203, Ferrara, 44121 Italy; 2 Operative Unit of Dentistry, AUSL of Ferrara, Ferrara, Italy; 3 Department of Life Sciences and Biotechnology, University of Ferrara, Ferrara, Italy

**Keywords:** Periodontal pocket, Periodontitis, Prognosis, Regenerative medicine, Minimally invasive surgical procedures

## Abstract

**Objectives:**

to evaluate the association between patient- and local- factors and pocket closure (i.e., probing depth, PD, ≤4 mm) following surgical treatment of intraosseous defects with the Single Flap Approach (SFA).

**Materials & methods:**

a retrospective analysis of data from previous studies was conducted on 101 defects treated with SFA alone or in combination with enamel matrix derivative with/without a bovine-derived xenograft. Pocket closure at 12 months was the primary outcome. Age, sex, smoking status, baseline PD, tooth type, depth of the supraosseous component, radiographic depth of the intraosseous component, defect angle, defect morphology, treatment modality were considered as candidate determinants in a bivariate logistic regression analysis. Backward stepwise regression was used to identify the optimal set of factors significantly associated with pocket closure.

**Results:**

12-month pocket closure occurred in 74.3% of cases. The probability of pocket closure was significantly associated with baseline PD (OR = 0.741, 95%CI: 0.565–0.973; *p* = 0.031) and defect morphology, with defects classified as “mainly 1-wall” and “mainly 3-wall” showing greater odds for pocket closure compared to “mainly 2-wall” defects (OR = 7.125, *p* = 0.006; and OR = 5.225, *p* = 0.006, respectively).

**Conclusions:**

When performed according to the SFA, regenerative surgical procedures are associated with high probability of pocket closure at 12 months. Intraosseous lesions with deeper pre-surgery PD and/or prevalent 2-wall morphology have lower probability to be closed.

**Clinical relevance:**

Data from the present study may be of use to the clinician who wants to optimize the odds for pocket closure at a deep intraosseous lesion that is being approached according to the SFA.

**Supplementary Information:**

The online version contains supplementary material available at 10.1007/s00784-025-06396-0.

## Introduction

When periodontal pockets with probing depth (PD) ≥ 6 mm persist after steps I-II of periodontal therapy at sites with deep intraosseous defects, regenerative surgical therapy is recommended. In such scenario, the use of flaps that maximize interdental soft tissue preservation in combination with regenerative devices was identified as the most indicated strategy [1, 2].

Recently, *pocket closure* (i.e., PD ≤ 4 mm) either alone or in combination with the presence of bleeding on probing (BoP) has been proposed as a patient-related endpoint of active periodontal therapy [3]. In this respect, pocket closure has been recently combined with the magnitude of Clinical Attachment Level (CAL) gain to generate a Composite Outcome Measure (COM) that may be of use to report the outcomes of periodontal regenerative treatment [4]. Based on a retrospective analysis of defects evaluated with COM at 6 months after surgery and followed for an average of 4 years, defects with a closed pocket at 6 months showed higher odds for CAL stability, higher tooth survival and less need for surgical re-interventions compared to those with residual PD > 4 mm [5]. Consistently, the key role of pocket closure for the stability of regenerative outcomes has been documented even in studies with a follow-up of 20 years [6].

Based on the results of a recent systematic review [7], the rate of pocket closure ranged from 71.4 to 100% when defects are treated with a regenerative strategy. Considering this variability, the identification of the determinants of pocket closure may be considered of clinical relevance. Therefore, the aim of the present study was to evaluate the association of patient- and defect-related factors and pocket closure following surgery based on the *Single Flap Approach* (SFA) [8, 9] with/without regenerative devices.

## Methods

### Experimental design and ethical aspects

The study is a retrospective analysis of data from previous trials on the SFA [10–13]. All trials had previously received approval from the Local Ethical Committee (Comitato Etico Area Vasta Emilia Centro, CE-AVEC, protocol numbers: #67; #151290; #170792). This study followed the Strengthening the Reporting of Observational Studies in Epidemiology (STROBE) Guidelines.

### Study population

Data were extracted from the record charts of patients receiving periodontal treatment and part of study cohorts included in previous trials assessing the clinical performance of regenerative procedures based on SFA [10–13]. All surgeries followed a strict surgical and post-surgical protocol (see paragraph on Clinical Procedures) and were performed by experienced periodontal surgeons (L.T., R.F., L.M).

Defects were included in the present analysis if: (i) undergoing buccal/oral SFA alone or in association with enamel matrix derivative (EMD) with or without deproteinized bovine bone mineral (DBBM) for the treatment of a residual ≥ 6 mm pocket associated with an intraosseous defect at least 3 mm deep (as evaluated radiographically and confirmed intraoperatively); (ii) all clinical and radiographic data related to the pre-surgery visit (baseline) and the 1-year follow-up (see the paragraph “*Study parameters*” for details) were available. Grade 2 and 3 furcation involvement [14] or inadequate endodontic treatment of the tooth presenting the intraosseous defect determined defect exclusion from the study.

Patients were included in the present analysis if: (i) full-mouth plaque score (FMPS [15]) and full-mouth bleeding score (FMBS [16]) < 20% were recorded at the time of the surgical procedure; and (ii) compliant with the scheduled post-surgical recall sessions for professional plaque removal.

Each patient contributed the study with one defect. For patients with multiple eligible defects, therefore, only the defect with the greater pre-surgery CAL was selected for inclusion in the present analysis. If two or more defects within the same patient had the same pre-surgery CAL, one defect was chosen randomly.

### Clinical procedures

#### Surgical procedures

Each intraosseous defect was accessed with buccal or oral SFA, as previously described [8, 9]. Briefly, a sulcular incision was performed, keeping the mesio-distal flap extension limited while ensuring proper access for defect debridement. An oblique or horizontal butt-joint incision was performed at the base of the interdental papilla overlying the defect. The greater the distance from the tip of the papilla to the underlying bone crest, the more apical (i.e., closer to the base of the papilla) the incision in the interdental area. A buccal/oral mucoperiosteal envelope flap was elevated using a microsurgical periosteal elevator, leaving the residual portion of the interdental supracrestal soft tissues undetached. The root was debrided by ultrasonic device (Insert 1 S, Piezosteril 5; Castellini S.p.A., Castel Maggiore, Bologna, Italy) and the defect was degranulated using Hirschfeld file scalers. SFA was performed alone or in addition to EMD with/without DBBM. The adjunctive use of DBBM was selected for wide, mainly 1–2 wall defects located at posterior teeth to optimize the regenerative outcome [17] as well as for defects (irrespective of configuration and severity) located at esthetically-sensitive areas to mitigate the post-surgery gingival recession [10]. Primary flap closure was obtained by a specific suturing technique as previously described [9].

#### Post-surgery procedures

Sutures were removed at 2 weeks after surgery. The patients were asked to abstain from mechanical oral hygiene procedures in the surgical area for 2 weeks. A 0.12% chlorhexidine mouth rinse (10 mL twice per day for 3–4 weeks) was prescribed to support local plaque control. Each patient was enrolled in a monthly recall program for 3 months and then recalled for oral hygiene reinforcement and supra- and iuxta-gingival professional mechanical plaque removal [18–20] at 6-, 9-, and 12-months post-surgery. Defects have been clinically and radiographically re-evaluated at 12 months following surgery. After the 12-month re-evaluation, patients were enrolled in a supportive periodontal care program tailored according to the PerioRisk level [18–20].

### Study parameters

The following study parameters, as evaluated immediately before the surgical procedure (baseline), were extracted from the clinical record charts:

- age (in years);
- biological sex (male, female);
- smoking status (non-smoker/current smoker);
- tooth type (single-root/ multi-root);
- CAL, measured (in mm) from the CEJ or the apical margin of a restoration to the bottom of the pocket;
- PD, measured (in mm) from the gingival margin to the bottom of the pocket;
- Gingival recession (REC), measured (in mm) from the CEJ or the apical margin of a restoration to the gingival margin.

PD was also re-assessed at 12-month post-surgery. Accordingly, a pocket was considered as “closed” if a residual PD ≤ 4 mm was recorded.

The following intra-surgical parameters, assessed immediately after the completion of root and defect debridement, were also extracted from record charts: (i) depth of the intraosseous component (IBD), measured as the distance between the most coronal point of the alveolar crest and the base of the defect; (ii) defect morphology (i.e., number of residual bony walls), measured as “mainly 1-wall”, “mainly 2-wall”, and “mainly 3-wall”; (iii) treatment modality (i.e., no biomaterials, EMD, EMD + DBBM).

Radiographic measurements were assessed on non-standardized periapical radiographs taken at baseline by an experienced and calibrated examiner (A.S.) using a dedicated software (NIS Elements™; Nikon Instruments S.P.A. Campi Bisenzio, Firenze, Italy). Radiographic depth of the intraosseous component (r_INTRA) was assessed as the linear distance (in mm) between the most apical extension of the defect (i.e., where the periodontal ligament space was considered having a normal width) and the bone crest of the adjacent tooth, as projected on the root surface of the tooth associated to the intraosseous defect. Defect angle was also assessed (in degrees) [21].

Trained clinical operators, with prior experience in SFA clinical trials and interclass correlation coefficients exceeding 0.8 for single measurements, performed all clinical and radiographic assessments.

### Statistical analysis

A statistical software (STATISTICA– v7.1 StatSoft GmbH, Germany) was used for data analysis. Patient was regarded as the statistical unit. Descriptive statistics included frequency analysis for categorical variables and mean ± standard deviation (SD) for continuous variables. The closed pocket (i.e. 12-month PD ≤ 4 mm) was regarded as the primary outcome of the study. Candidate determinants of pocket closure were selected among those variables collected during routine clinical examination [22, 23] with an expected or documented impact on the outcomes of periodontal regenerative therapy. Candidate variables were: age, sex, smoking status, baseline PD, tooth type, depth of the supraoseous component of the defect (SUPRA), r_INTRA, defect angle, defect morphology, treatment modality. SUPRA was calculated, in mm, as [(baseline PD-IBD)-baseline REC] [24]. To assess their impact on pocket closure, all factors were initially tested in a bivariate logistic regression analysis. To facilitate a more immediate understanding of the effects of continuous quantitative regressors on the outcome variable, the logistic regression coefficients for these variables were transformed into odds ratios (ORs) using the exponential function. These ORs, along with those estimated for categorical variables, were reported in the related table. In order to identify the optimal set of variables for inclusion in the multivariate model, a backward stepwise regression was applied with the objective of maintaining the model as straightforward as possible while also ensuring the highest predictive power. The final model included only significant factors (*p* < 0.05).

## Results

### Study population

One-hundred and one patients, each contributing one intraosseous defect, were included for analysis. Patient and defect characteristics are reported in Table 1. Non-smokers were more prevalent than current smokers. Three-, 2- and 1-wall defects accounted for 37.6%, 42.6%, and 19.8% of the population, respectively. SFA in combination with EMD + DBBM was the most frequent strategy used (47.5%), followed by SFA alone (34.7%) or in combination with EMD (17.8%).

**Table 1:** Patient and defect characteristics, as recorded pre- and intra-surgically

PARAMETER	DESCRIPTIVE STATISTICS
***Patient-related characteristics (n = 101)***	
Age (years) *mean ± SD (min*,* max)*	54.1 ± 9.4 (36.0–78.0)
Biological Sex Males n *(%*) Females n *(%*)	56 (55.4) 45 (44.6)
Smoking status Current Smokers n *(%*) Non-smokers n *(%*)	14 (13.9) 87 (86.1)
***Defect-related characteristics (n = 101)***	
Tooth type Single-root n *(%*) Multi-root n *(%*)	77 (76.2) 24 (23.8)
Baseline PD (mm) *mean ± SD (min*,* max)*	8.0 ± 1.8 (5.5–15.0)
r_INTRA (mm) *mean ± SD (min*,* max)*	5.3 ± 2.0 (2.6–13.8)
Defect angle (degrees) *mean ± SD (min*,* max)*	36.3 ± 11.0 (14.3–67.7)
SUPRA (mm) *mean ± SD (min*,* max)*	4.0 ± 1.6 (2.0–14.0)
Defect morfology 1-wall n *(%*) 2-wall n *(%*) 3-wall n *(%*)	20 (19.8) 43 (42.6) 38 (37.6)
Treatment modality SFA alone n *(%*) SFA + EMD* n *(%*) SFA + EMD*+DBBM^§^ n *(%*)	35 (34.7) 18 (17.8) 48 (47.5)

* Emdogain gel, Institute Straumann, Basel, Switzerland

^§^ Bio-Oss spongiosa granules, 0.25–1.0 mm, Geistlich Pharma, Wolhusen, Switzerland

### Clinical outcomes

Patient distribution according to PD, as assessed at baseline and 12 months following surgery, is reported in Table 2. Treatment resulted in 74.3% of patients with closed pockets while 25.7% of patients showed a persistent 5-mm pocket. CAL, PD, and REC recorded at baseline and 12 months following surgery with related changes are shown in Table S1. A mean CAL gain of 3.3 ± 1.7 mm (*p* < 0.001) associated with a PD reduction of 4.2 ± 1.7 mm (*p* < 0.001) were observed. A significant increase in REC of 1.0 ± 1.2 mm was also recorded. r_INTRA decreased from 5.3 ± 2.0 mm at baseline to 1.9 ± 1.7 mm at 12 months.

**Table 2:** Patient distribution according to PD, as assessed at baseline and 12 months following surgery

PD (mm)	baseline *n* (%)	12-month *n* (%)
**2**	0 (0)	5 (5.0)
**3**	0 (0)	34 (33.7)
**4**	0 (0)	36 (35.6)
**5**	0 (0)	26 (25.7)
**6**	16 (15.8)	0 (0)
**7**	30 (29.7)	0 (0)
**8**	22 (21.8)	0 (0)
**9**	16 (15.8)	0 (0)
**10**	7 (6.9)	0 (0)
**11**	6 (5.9)	0 (0)
**12**	1 (1)	0 (0)
**13**	2 (2.0)	0 (0)
**14**	0 (0)	0 (0)
**15**	1 (1)	0 (0)

### Influence of candidate determinants on the probability of 12-month pocket closure

The results of the bivariate logistic regression model (Table S2) showed that r_INTRA and baseline PD were significantly associated with the probability for pocket closure with an odd ratio (OR) of 1.294 (95%CI: 1.035 to 1.617; *p* = 0.024) and 1.387 (95%CI: 1.067 to 1.802; *p* = 0.015), respectively.

In the stepwise multivariate regression final model (Table 3), baseline PD and defect morphology were the only factors that significantly influenced the probability of pocket closure. Specifically, the probability for pocket closure was negatively associated with baseline PD (OR of 0.741, 95%CI: 0.565 to 0.973; *p* = 0.031), while defects classified as “mainly 1-wall” and “mainly 3-wall” had significantly greater odds for pocket closure compared to “mainly 2-wall” defects, with the respective ORs being 7.125 (95%CI: 1.674 to 30.329 *p* = 0.006) and 5.225 (95%CI: 1.774 to 15.390; *p* = 0.002).

**Table 3:** Stepwise multivariate regression final model for pocket closure (12-month PD ≤ 4 mm)

(A) Variables	Df	Wald statistics	*p*-value
Constant	1	10.380	0.001
Defect morphology	2	10.596	**0.005**
Baseline PD	1	4.639	**0.031**
(B) **Variables**	***OR***	**95% CI**	***p*** **-value**
Baseline PD	0.741	0.565–0.973	0.031*
Defect morphology			
1-wall vs. 2- wall	7.125	1.674 to 30.329	0.006*
1-wall vs. 3 - wall	1.364	0.240 to 7.750	0.402
3-wall vs. 2 - wall	5.225	1.774 to 15.390	0.002*

* The final model included only significant factors (*p* < 0.05)

## Discussion

The present retrospective study aimed at identifying patient-related and local factors that influence the achievement of pocket closure after surgical therapy of deep intraosseous defects. One hundred and one defects treated with the SFA either alone or associated with regenerative biomaterials (EMD or EMD plus DBBM) were evaluated for pocket closure (defined as PD ≤ 4 mm) at the 12-month follow-up visit. The results of the stepwise multivariate regression model showed that the probability of achieving pocket closure is significantly influenced by baseline PD and defect morphology.

Despite the definition of periodontitis treatment endpoints provided by the S3 level Clinical Practice Guideline of the European Federation of Periodontology [1] is based on residual PD and bleeding on probing (BoP), our analysis focused exclusively on pocket closure due to the lack of data on BoP in some patient record charts. It must be considered, however, that a robust correlation between PD and BoP is widely documented at both site- and patient-level [25–29]. Based on data from a large patient cohort with heterogeneous periodontal conditions, the estimated probability for a site to be BoP-positive is 18% and 30% for sites with PD = 3 mm, and PD = 4 mm, respectively, while it markedly increases to 46% and almost 60% for PDs of 5 mm and 6 mm, respectively. When PD > 9 mm, the probability approaches 100% [26]. Therefore, the strong dependence of BoP on PD seems to mitigate the absence of BoP as a limitation of our study. Consistently, other authors considered PD (and not BoP) to propose new clinical endpoints for periodontitis treatment [3] and regenerative periodontal therapy [4].

In the present material, pocket closure was achieved in almost 75% of defects. This proportion falls within the range of 71.4-100% that was reported in a recent systematic review [7] including 12 RCTs on periodontal regenerative therapy, and further supports the need for the identification of determinants of pocket closure as attempted in the present analysis. Among studies included in the review [7] and reporting the highest pocket closure rate, a clinical trial where a variant of the SFA (i.e., the modified minimally-invasive surgical technique, M-MIST [30]) was used either alone or in combination with EMD or EMD plus DBBM obtained 100% closed pockets at 12 months [31]. The higher pocket closure reported by Cortellini et al. [31] compared to our study may be partly due to optimized wound healing conditions provided by M-MIST, the latter being characterized by minimal mesio-distal flap extension. Based on the negative association between pre-surgery PD and pocket closure rate that was observed in either the present analysis or other clinical trials evaluating different treatment strategies for pocket closure [32–36], however, it is also plausible that differences in baseline PD may have partly accounted for differences in pocket closure rates between studies. In this respect, mean baseline PD of the present patient cohort was 8.1 mm, while ranged from 7.3 mm to 7.8 mm (depending on the treatment group) in the study by Cortellini et al. [31].

In our analysis, mainly 3-wall and mainly 1-wall defects showed higher probabilities of achieving pocket closure compared to mainly 2-wall defects (OR = 7.1 and OR = 5.2, respectively). This finding is not fully consistent with classical and recent studies indicating that the number of residual bony walls is positively associated with treatment outcomes (including PD reduction/pocket closure) [37–39]. A first, plausible explanation can be extrapolated from the distribution of treatment modalities (which were selected at operator discretion) within each category of defect morphology (Table S3). The dominant prevalence (60%) of EMD + DBBM treatment in mainly 1-wall defects probably reflects the intention of the operators to compensate with regenerative devices the negative impact of the limited number of residual defect walls on SFA outcomes, as previously demonstrated [17, 40]. The rather homogeneous distribution of treatment modalities within the other two defect morphologies, which probably determined the high level of statistical independency (77%; data not shown) of treatment modality from defect morphology, should be interpreted differently for each defect type. For mainly 3-wall defects, it probably reveals the awareness of the operators that such lesions are characterized by high intrinsic healing potential [31, 41] and benefit from the combination of a bioactive agent and a graft material only if the minimization of interproximal REC is a priority [10]. For mainly-2 wall defects, it probably reflects the discretion of the operator in absence of clear recommendations from the literature on SFA, with heterogeneity in treatment selection penalizing treatment outcomes in this specific category. When considering that most of the investigated candidate determinants were related to the hard tissue component (e.g., defect angle, severity of the supraosseous and intraosseous components) and showed no sufficient statistical robustness in the stepwise multivariate regression analysis, a second hypothesis to explain the lower pocket closure rates of mainly 2-wall defects compared to mainly 1-wall defects may reside in the role of other local factors related to supracrestal soft tissues that were not considered in the present analysis. Among these, the presence of interdental contact point, papillary width and the presence of interdental soft tissue craters were previously shown to influence early wound healing following SFA [11] and probably deserve further investigation for their potential effect on pocket closure at later timepoints.

The robust evidence supporting the negative impact of smoking on the outcomes (including pocket reduction) of periodontal regenerative procedures [37–39] makes the observed lack of effect of smoking in this study particularly unexpected. However, this finding can be considered in the light of the limited number of smokers (13.8% of the study population) and their moderate to low daily exposure (< 10 cigarettes per day). In this respect, no significant inter-group differences in treatment outcomes were previously reported for light smokers (i.e., patients consuming < 10 cigarettes/day) and non-smokers undergoing SFA in association with EMD plus DBBM [13]. Overall, these findings support the possibility that the combination of the biological advantages of the SFA [40] and EMD [41–44] may compensate the detrimental effect of light smoking on 12-month pocket closure.

The present findings should be interpreted in the light of some potential study limitations beyond those mentioned in the previous paragraphs. The absence of diabetic patients prevented the possibility to clarify the potential impact of diabetes on pocket closure, as recent studies have produced conflicting findings [45, 46]. Moreover, the lack of information on FMPS at the 12-month follow-up visit precludes the possibility to evaluate the impact of supragingival plaque control on treatment outcomes, including pocket closure. Since patients were enrolled in a stringent post-surgical recall program, however, it is reasonable to assume that low levels of full-mouth plaque deposits and periodontal inflammation have been maintained throughout the follow-up period.

## Conclusions

In conclusion, the results of the present study showed that regenerative surgical procedures performed according to the SFA are associated with high probability (almost 75%) of pocket closure at 12 months. Intraosseous lesions with deeper pre-surgery PD and/or prevalent 2-wall morphology have lower probability to exhibit pocket closure following surgery.

## Electronic supplementary material

Below is the link to the electronic supplementary material.

Supplementary Material 1

## Data Availability

No datasets were generated or analysed during the current study.
