# Supplementary material for: Factors influencing pocket closure in surgically-treated intraosseous defects. A retrospective analysis
Source: Clin Oral Investig. 2025 May 28;29(6):316. doi: 10.1007/s00784-025-06396-0 (PMC12119774; doi:10.1007/s00784-025-06396-0)
Supplement: Supplementary file 1 — Supplementary Material 1 [file 784_2025_6396_MOESM1_ESM.docx]

**SUPPLEMENYARY TABLES**

**Table S1**. Clinical and radiographic measurements at baseline and 12 months following surgery.

|  | **Baseline** | **12-months** | **Change** | ***p*** |
| --- | --- | --- | --- | --- |
| **CAL (mm)**  *mean±SD (min-max)* | 9.6 ± 2.1  (5.5-17.0) | 6.3 ± 1.8  (2.0-11.0) | 3.3 ±1.7  (-1.0-9.0) | < 0.001 |
| **PD (mm)**  *mean±SD (min-max)* | 8**.**1 ± 1.8  (5.5-15.0) | 3.8 ± 0.9  (2.0-5.0) | 4**.**2 ±1.7  (1.0-10.0) | < 0.001 |
| **REC (mm)**  *mean±SD (min-max)* | 1.6 ± 1.5  (0-8.0) | 2.5 ± 1.6  (0-7.0) | -1.0±.1.2  (-6.0-1.0) | < 0.001 |
| **r_INTRA (mm)**  *mean±SD (min-max)* | 5.3 ± 2.0  (2.6-13.8) | 1.9 ± 1.7  (0-8.3) | 3.4 ± 2.1  (-1.6-10) | < 0.001 |

**Table S2.** Bivariate logistic regression model for pocket closure (i.e.,12-month PD≤4mm).

| **Variables** | ***OR*** | **95% CI** | ***p*-value** |
| --- | --- | --- | --- |
| ***Patient-related*** |  |  |  |
| Age | 1.022 | 0.972 to 1.074 | 0.391 |
| Sex | 1.744 | 0.683 to 4.454 | 0.242 |
| Smoking status | 0.846 | 0.237 to 3.018 | 0.795 |
|  |  |  |  |
| ***Defect-related*** |  |  |  |
| r_INTRA | 1.294 | 1.035 to 1.617 | **0.024*** |
| Defect angle | 0.973 | 0.931 to 1.016 | 0.212 |
| SUPRA | 1.180 | 0.893 to 1.558 | 0.242 |
| Defect morphology | 1.168 | 0.634 to 2.149 | 0.615 |
| Tooth type | 1.639 | 0.596 to 4.507 | 0.335 |
| Treatment modality | 1.239 | 0.750 to 2.045 | 0.399 |
| Baseline PD | 1.387 | 1.067 to 1.802 | **0.015*** |

(*) significant at 5% level.

**Table S3.** Distribution of defects across various treatment modalities (SFA, SFA+EMD, SFA+EMD+DBBM) according to the number of residual bony walls.

|  | **SFA** | **SFA+EMD** | **SFA+EMD+DBBM** |
| --- | --- | --- | --- |
| 1-WALL DEFECTS  (n=20) | **5**  (25%) | **3**  (15%) | **12**  (60%) |
| 2-WALL DEFECTS  (n=43) | **16**  (37.2%) | **7**  (16.2%) | **20**  (46.6%) |
| 3-WALL DEFECTS  (n=38) | **14**  (36.8%) | **8**  (21.1%) | **16**  (42.1%) |
